# RNA-Seq Identifies Marked Th17 Cell Activation and Altered CFTR Expression in Different Atopic Dermatitis Subtypes in Chinese Han Populations

**DOI:** 10.3389/fimmu.2021.628512

**Published:** 2021-04-01

**Authors:** Xin Tian, Baoyi Liu, Lijie Chen, Yongyi Xie, Jingyao Liang, Yan Yang, Lei Shao, Jing Zhang, Jianqin Wang, Xibao Zhang, Zhouwei Wu, Yumei Liu

**Affiliations:** ^1^ Department of Dermatology, Guangzhou Institute of Dermatology, Guangzhou, China; ^2^ Department of Dermatology, Shanghai General Hospital, Shanghai Jiao Tong University School of Medicine, Shanghai, China; ^3^ Department of Dermatology, Affiliated Shenzhen Maternity and Child Healthcare Hospital, Southern Medical University, Shenzhen, China

**Keywords:** atopic dermatitis, extrinsic AD, intrinsic AD, heterogeneity, atopic march

## Abstract

**Background:**

Patients with atopic dermatitis (AD) exhibit phenotypic variability in ethnicity and IgE status. In addition, some patients develop other allergic conditions, such as allergic rhinitis (AR), in subsequent life. Understanding the heterogeneity of AD would be beneficial to phenotype-specific therapies.

**Methods:**

Twenty-eight Chinese AD patients and 8 healthy volunteers were enrolled in the study. High-throughput transcriptome sequencing was conducted on lesional and nonlesional skin samples from 10 AD patients and matched normal skin samples from 5 healthy volunteers. Identification of differentially expressed genes (DEGs), KEGG pathway analyses, and sample cluster analyses were conducted in the R software environment using the DEseq2, ClusterProfiler, and pheatmap R packages, respectively. qRT-PCR, Western blotting, and ELISA were used to detect gene expression levels among subtypes. Correlation analysis was performed to further investigate their correlation with disease severity.

**Results:**

A total of 25,798 genes were detected per sample. Subgroup differential expression analysis and functional enrichment analysis revealed significant changes in the IL17 signaling pathway in Chinese EAD patients but not in IAD patients. DEGs enriched in cytokine-cytokine receptor interactions and gland secretion were considered to be associated with atopic march. Further investigations confirmed a marked IL17A upregulation in Chinese EAD with a positive relationship with total IgE level and AD severity. In addition, increased IL17A in AD patients with AR demonstrated a closer association with AR severity than IL4R. Moreover, AQP5 and CFTR were decreased in the lesions of AD patients with AR. The CFTR mRNA expression level was negatively associated with the skin IL17A level and AR severity.

**Conclusion:**

Our research characterized marked Th17 activation in Chinese EAD patients, and altered expression of IL17A, IL4R, AQP5, and CFTR in AD patients with AR was associated with AR severity. It partially explained the phenotypic differences of AD subtypes and provided potential references for endotype-targeted therapy.

## Introduction

Atopic dermatitis (AD) is characterized by pruritus with a chronic course of exacerbation and remission (1, 2). Patients with AD have a high propensity to develop other allergic conditions, such as asthma, allergic rhinitis (AR), and food allergies, which is called “atopic march” (AM) (2, 3). The clinical signs of AD often predate the development of atopic comorbidities, suggesting that AD might be an “entry point” for subsequent allergic diseases (3).

Recent studies have provided insights into the heterogeneity of AD among ethnicities and subtypes (1). It was reported that susceptibility loci associated with AD were only partially overlapped among different ethnicities by Genome-wide association study (GWAS) (4, 5), and Asian patients showed characteristic remarkable Th17 axis activation (1, 6). Besides, it is widely accepted that AD can be divided into two subtypes based on lgE levels (1, 2). Patients with high total and environmental serum IgE levels are diagnosed with extrinsic AD (EAD), which is the predominant subtype (accounting for 80%). This subtype has a close relationship with filaggrin mutation, personal and family atopic background, eosinophilia, and tendency to develop AR or asthma (1, 7). Intrinsic AD (IAD) is characterized by normal IgE levels, the absence of specific IgE for environmental and food allergens, less barrier function damage, and more similar to allergic contact dermatitis (2, 7, 8). Although the molecular endotypic difference between EAD and IAD has been reported by several studies (1, 2, 7), ethnic factors were not taken into account. Given AD heterogeneity exists across ethnicities and subtypes, previous studies based on a single aspect may result in inaccurate conclusions.

High-throughput sequencing allows the analysis of the global transcriptome and accurate detection of gene expression on a larger scale (9). To characterize the heterogeneity with precise stratifications, we performed high-throughput RNA-seq on lesional and nonlesional skin samples from 10 AD patients and normal skin samples from 5 healthy volunteers and analyzed the difference between the IAD and EAD and the genes related to the AM in the Chinese Han population. Our results showed that a marked Th17 activation in EAD rather than IAD. In addition, IL17A, IL4R, AQP5, and CFTR were associated with AM in the Chinese Han population.

## Materials and Methods

### Patients Enrollment and Samples Collection

Twenty-eight Chinese Han AD patients and 8 age- and sex-matched healthy volunteers were recruited with signed consent obtained. Among all participants, 19 subjects were from Guangzhou, and 17 were from Shanghai, representing different regions in China. The enrollment criteria for patients were as follows: (1) Met Williams diagnostic criteria clinically and the main characteristics of AD pathologically; (2) SCORAD scores ≥ 25; and (3) did not receive any treatment in the past two weeks. The subjects were divided into two groups (10 IAD patients and 18 EAD patients) based on raised levels of IgE (> 500 kU/L) and/or personal or familial history of atopy (8). In these 28 AD patients, 15 patients had AR, 11 patients did not have AR, and the condition of 2 patients was unknown. In those 8 healthy volunteers, 3 volunteers had AR, and 5 volunteers did not have AR. The total nasal symptom score (TNSS) was used to measure the severity of AR (10). The study was approved by the local ethics committee and conducted according to the principles of the Declaration of Helsinki. A 4 mm-diameter skin biopsy was taken from lesional and nonlesional skin in every AD patient and in 8 healthy volunteers. Samples were stored at −80°C until processing for extraction of RNA and protein. The information for the participants is shown in **Supplementary Table S1**. 

### RNA-Seq

RNA-seq was conducted on nonlesional and lesional skin samples from 10 patients (P1-P10) and 5 healthy skin samples (H1-H5). Sample details of RNA-seq are shown in **Supplementary Table S2**. Total RNA was extracted using TRIzol (Invitrogen, USA) following the manufacturer’s instructions. The total RNA concentration, the RIN value, 28S/18S, and the fragment size were measured using an Agilent 2100 Bioanalyzer (Agilent, USA). The BGISEQ-500 (Shenzhen Huada Gene, China) platform based on sequencing by synthesis (SBS) technology was used for high-throughput sequencing to obtain a 100 bp sequencing read. The raw data were subjected to quality control to obtain effective reads. SOAPnuke (v1.4.0) and Trimmomatic (v0.36) were used to perform statistical analysis and filter out reads of low to moderate quality, polluted connectors, and unknown nucleotides with high N content before data analysis to ensure reliability. 

### RNA-Seq Analyses

RNA-seq analyses were conducted in the R environment (v3.6.2). The DEseq2 R package was used to identify the differentially expressed genes (DEGs) (|log2FC| ≥ 1, adj. P ≥ 0.05) between each group following the previously described methods (11). After constructing appropriate gene sets, two types of enrichment analyses were conducted: overrepresentation analysis (ORA) and gene set enrichment analysis (GSEA) using the ClusterProfiler R package (12). Sample cluster analysis of the DEGs was performed using the pheatmap R package (13). Annotation of the genes and pathways was provided by the KEGG (Kyoto Encyclopedia of Genes and Genomes) database (http://www.genome.jp/kegg). Only KEGG pathways enriched at *P* < 0.05 were considered. The workflow of the RNA-seq analyses is shown in **Supplementary Figure S1**.

### Microarray Analysis

Three microarray studies from the Gene Expression Omnibus (GEO) database were utilized, including GSE75890, GSE146352, and GSE124700. GSE75890 included 9 IAD, 5 EAD, and 8 normal skin (HS) samples from Denmark to analyze the heterogeneity between IAD and EAD in patients from Europe. In brief, the raw data were downloaded from the repository. DEGs (|log2FC| ≥1, adj. P ≤ 0.05) between each group were identified by the limma R package, and KEGG pathway enrichment was conducted by the methods described above in the R environment. GSE146352 and GSE124700 were conducted by the same platforms, including 4 AD, 3 AM, and 3 HS skin samples from Korea in total. The two datasets were merged as a microarray of AM and mined for the expression levels of AM-associated genes involved in the IL17 signaling pathway and gland secretion.

### Quantitative Real-Time RT-PCR (qRT-PCR)

Total RNA was extracted using TRIzol (Invitrogen, USA) following the manufacturer’s instructions and transcribed into cDNA using PrimeScript^TM^ RT Master Mix (Takara, Japan). The relative expression of various target genes was determined by RT-PCR using TB Green® Premix Ex Taq™ II (Takara, Japan). Gene expression was normalized to that of *GAPDH*. The primer sequences used in this study are listed in **Supplementary Table S3**.

### ELISA Assessment

The IL17A and IL4R proteins were measured by ELISA (ab216167 and ab243668, Abcam, USA). Briefly, standards and samples were added into the wells and incubated with antibody cocktails for 1 h at room temperature on a plate shaker set to 400 rpm. Then, aspirate and wash each well with 350 µL of 1X wash buffer three times. After washing, 100 µL of TMB development solution was added to each well and further incubated for 10 min in the dark on a plate shaker set to 400 rpm. Finally, 100 µL of stop solution was added to each well and shaken for 1 min, and then the intensity of the color was measured at 450 nm.

### Western Blotting

Frozen skin samples were cut into pieces and lysed with ice-cold radioimmunoprecipitation (RIPA) lysis buffer containing protease inhibitors (Beyotime, China) to obtain tissue homogenates. The protein concentration of each sample was detected using a bicinchoninic acid (BCA) kit (Beyotime, China). After normalization to PBS, the lysates were mixed with loading buffer and incubated for 10 min at 95°C. Approximately 20 µg of protein from each sample was separated by 10% SDS-PAGE and transferred to PVDF membranes (Merck Millipore, USA). After blocking in 5% bovine serum albumin (Yeasen, China) for 1 h, the membranes were incubated with primary antibodies overnight at 4°C and with secondary antibodies for 1 h. GAPDH and β-tubulin were used as loading controls. Bound antibodies were detected using the ECL Western blot detection system (Merck Millipore, USA) and quantified by ImageJ (National Institutes of Health, USA). The information for the primary antibodies used in the experiment is listed in **Supplementary Table S4**.

### Statistical Analysis

Statistical analysis of RNA-seq was performed by using R software (www.R-project.org) and packages available through Bioconductor (www.bioconductor.org). The experimental data are presented as the mean ± SD and were analyzed using unpaired Student’s *t*-test and one-way ANOVA followed by Tukey’s or Dunnett’s multiple comparisons posttest. Correlations were determined by Pearson correlation analysis. All analyses of experimental data and clinical features were performed using GraphPad Prism 7 software (San Diego, USA). Differences were regarded as significant at *P* < 0.05. 

## Results

### Significant Alterations in Focal Adhesion and the NOD-Like Receptor Signaling Pathway in AD Skin From the Chinese Han Population

A total of 25 skin samples, including 10 lesional (L) and 10 nonlesional skin samples (NL) from AD patients and 5 HS from healthy volunteers, were subjected to RNA-seq. Through RNA-seq, we generated 107 ± 7 million (mean ± SD) reads per sample, with reads averaging 100 bp in length, producing 11 Gb of cDNA sequence per sample. After mapping the RNA-seq reads to the reference genome, we obtained a high mapping rate of 85% on average. A total of 25,798 genes were detected, including 19,483 annotated genes.

**Figure 1:**
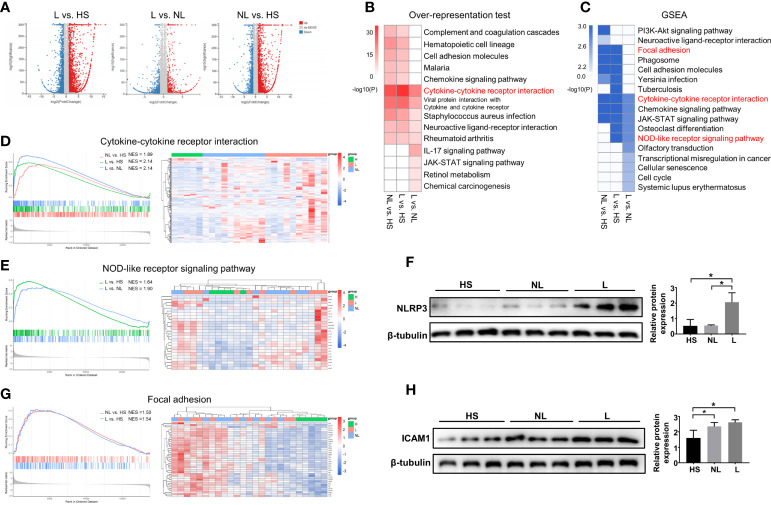
DEG identification and KEGG enrichment pathway analyses between the HS and NL groups, HS and L groups, and NL and L groups. **(A)** Volcano plot of DEGs between the L and HS groups, the L and NL groups, and the NL and HS groups identified by differential expression analyses. HS group (n = 5), NL group (n = 10), and L group (n = 10). **(B)** Top 10 enriched KEGG pathways analysis by overrepresentation test and **(C)** by GSEA among the three groups. (*P* < 0.05) The GSEA enrichment plot and the sample cluster analyses based on the expression pattern of involved DEGs of **(D)** cytokine-cytokine receptor interaction, **(E)** NOD-like receptor signaling pathway, and **(G)** focal adhesion. Western blot and quantitative analysis of **(F)** NLRP3 and **(H)** ICAM1 among the HS (n = 3), NL (n = 3), and L (n = 3) groups. *P < 0.05 according to one-way ANOVA followed by Tukey's multiple comparisons.

Then, differential expression analyses between groups were performed using the DEseq2 R package. The results showed a total of 4924 DEGs between the L and HS groups, 4924 DEGs between the L and NL groups, and 4281 DEGs between the NL and HS groups (**Figure 1A**). KEGG analyses were employed by ORA and GSEA using the ClusterProfiler R package to explore changes that occurred in lesional and nonlesional skin. All the identified DEGs were subjected to KEGG analysis by ORA, and all annotated genes were input into GSEA. As shown in **Figures 1B, C**, significant changes in cytokine-cytokine receptor interactions were identified among the three groups by both ORA and GSEA. In addition, GSEA detected significant alterations in the NOD-like receptor signaling pathway in both the L vs. NL group and L vs. HS group, as well as changes in focal adhesion in both the L vs. HS group and NL vs. HS group. Sample cluster analyses were further performed to investigate the DEGs and/or core enriched genes involved in these three pathways. DEGs (or core enrichment genes) in the three pathways were upregulated in most samples from AD patients, especially in lesional skin (**Figures 1D, E, G**). As expected, the Western blotting results illustrated that NLRP3 was upregulated in lesional skin compared to nonlesional and healthy skin and was enriched in the NOD-like receptor signaling pathway (**Figure 1F**). The protein expression of ICAM1 involved in focal adhesion was increased in both nonlesional and lesional skin compared to healthy skin (**Figure 1H**). 

### Increased IL17A Characterizes Chinese EAD Patients Rather Than IAD Patients

**Figure 2:**
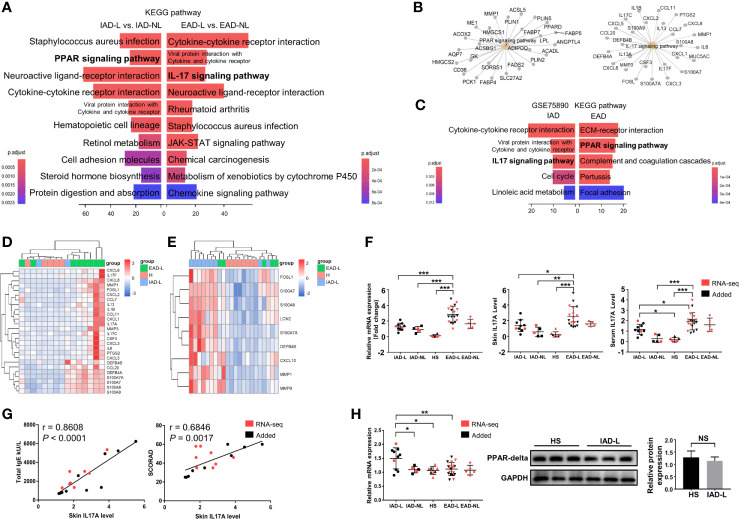
KEGG pathway enrichment analysis of DEGs in intrinsic and extrinsic AD. **(A)** Top 10 enriched KEGG pathways of the DEGs involved in Chinese IAD and EAD patients, *P* < 0.05. **(B)** The interaction networks of IAD- and EAD-specific pathways and the DEGs involved. **(C)** Top 5 enriched KEGG pathways of the DEGs involved in Denmark IAD and EAD patients, *P* < 0.05. **(D)** Sample cluster analyses based on the expression pattern of DEGs involved in the IL-17 signaling pathway in the Chinese Han population (HS group (n = 5), IAD-L group (n = 2), EAD-L group (n = 8)], and **(E)** in the Denmark population [HS group (n = 8), IAD-L group (n = 9), EAD-L group (n = 5)]. **(F)** The IL17A expression level in the skin and serum in the Chinese Han population [HS group (n = 8), IAD-L group (n = 10), IAD-NL group (n = 5), EAD-L group (n = 18), EAD-NL group (n = 5)]. **(G)** The correlation of skin IL17A levels with total IgE and SCORAD (n = 18). **(H)** The mRNA [HS group (n = 8), IAD-L group (n = 10), IAD-NL group (n = 5), EAD-L group (n = 18), EAD-NL group (n = 5)], protein expression level and quantitative analysis [HS group (n = 3), IAD-NL group (n = 3)] of PPARG. NS, no significance, *P < 0.05, **P < 0.01, ***P < 0.001 according to Student’s *t*-test analysis or one-way ANOVA followed by Tukey’s multiple comparisons.

To determine if there were specific pathways involved in different subtypes, differential expression analyses were performed between lesional and nonlesional skin samples from IAD patients (IAD-L vs. IAD-NL) and EAD patients (EAD-L vs. EAD-NL). A total of 2250 DEGs were identified in the IAD-L vs. IAD-NL group, as well as 1267 DEGs in the EAD-L vs. EAD-NL group (**Supplementary Figure S2**). The results of KEGG analyses revealed a remarkable change in the PPAR signaling pathway in IAD, while alterations in the IL17 signaling pathway were significant in EAD (**Figure 2A**). The interaction network of subtype-specific pathways and genes is shown in **Figure 2B**. We further performed differential expression analyses and enrichment analyses between each subtype and healthy skin samples from Denmark. Surprisingly, KEGG analysis results demonstrated a remarkable change in the IL17 signaling pathway in IAD but not EAD (**Figure 2C**). As shown in **Figures 2D, E**, an opposite expression pattern of genes enriched in the IL17 signaling pathway was identified between Chinese and Denmark AD patients.

We detected the mRNA and protein expression levels of IL17A and PPARG by qRT-PCR and Western blotting (or ELISA) to confirm the RNA-seq analysis results in the Chinese Han population. Consistently, the mRNA and protein expression levels of IL17A were significantly upregulated in the lesions and serum of EAD patients compared to the IAD and HS groups (**Figure 2F**). In addition, the correlation analysis revealed that skin IL17A levels were significantly and positively related to the total IgE level and AD severity (SCORAD) (**Figure 2G**). However, although the mRNA expression level of PPARG was increased in the IAD-L group compared to the HS group, its protein expression level showed no significant difference between the two groups (**Figure 2H**).

### Mild Immune Activation in Chinese IAD Patients

**Figure 3:**
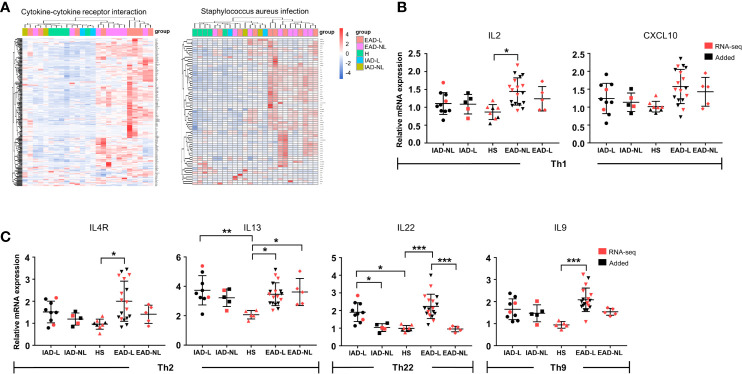
Cytokine levels in Chinese intrinsic and extrinsic AD patients. **(A)** Sample cluster analyses based on the expression pattern of DEGs involved in cytokine-cytokine receptor interactions and *Staphylococcus aureus* infection [HS group (n = 5), IAD-L group (n = 2), IAD-NL group (n = 2), EAD-L group (n = 8), EAD-NL group (n = 8)]. **(B, C)** The mRNA expression levels of cytokines involved in Th1, Th2, Th22, and Th9 cells in the skin [HS group (n = 8), IAD-L group (n = 10), IAD-NL group (n = 5), EAD-L group (n = 18), EAD-NL group (n = 5)]. *P < 0.05, **P < 0.01, ***P < 0.001 according to one-way ANOVA followed by Tukey’s multiple comparisons.

We further performed differential expression analyses and enrichment analyses of lesional (IAD-L vs. EAD-L) and nonlesional (IAD-NL vs. EAD-NL) skin samples. Similar to the above enrichment results, remarkable changes in cytokine-cytokine receptor interaction and *Staphylococcus aureus* infection were also identified in both groups (**Supplementary Figure S3**). As shown in **Figure 3A**, heatmaps and cluster analyses demonstrated that the expression level of DEGs in these two pathways was significantly upregulated in EAD, whereas the DEG expression pattern of IAD was more similar to those of healthy controls, indicating that immune activation in IAD was mild. To confirm these results, qRT-PCR was performed to investigate the mRNA levels of several cytokines. The results revealed that the expression levels of IL2 and IL9 were not obviously changed in IAD but were significantly increased in EAD. Elevated expression of IL4R, IL13, and IL22 was detected in both EAD and IAD (**Figures 3B, C**).

### Elevated IL17A and IL4R in Lesions Were Associated With Rhinitis Severity

**Figure 4:**
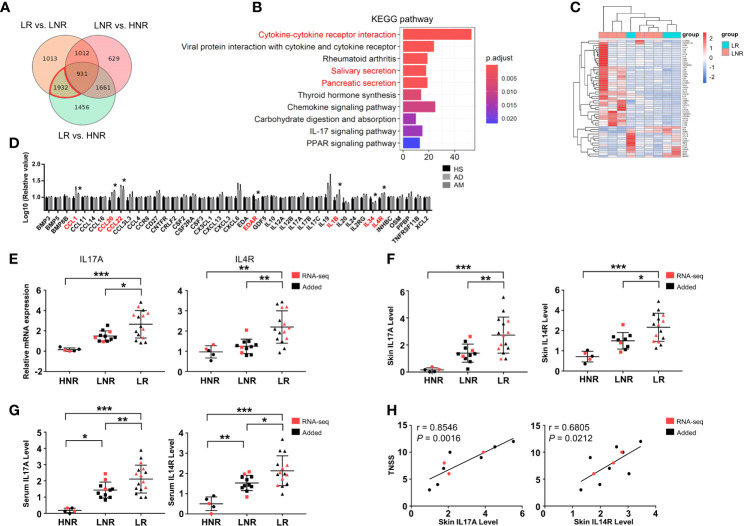
Aberrant cytokine identification in lesions of AD patients with atopic march. **(A)** Vann diagram showing the number of AM-associated genes in the red circle. **(B)** Top 10 enriched KEGG pathways, *P* < 0.05. **(C)** Sample cluster analyses based on the expression pattern of DEGs involved in cytokine-cytokine receptor interactions [LNR group (n = 3), LR group (n = 6)]. **(D)** The expression levels of DEGs enriched in cytokine-cytokine receptor interactions in the microarray of AM [HS group (n = 3), AD group (n = 4), AM group (n = 3)]. **(E)** The mRNA expression levels of IL17A and IL4R in the skin. **(F)** The protein expression levels of IL17A and IL4R in the skin and **(G)** serum [HNR group (n = 5), LNR group (n = 11), LR group (n = 15)]. **(H)** The correlation of skin IL17A and IL4R with TNSS (n = 11). *P < 0.05, **P < 0.01, ***P < 0.001 according to one-way ANOVA followed by Tukey’s or Dunnett’s multiple comparisons.

To understand the specific changes that occurred in AD patients with AR, differential expression analyses were performed among lesional skin samples from AD patients with AR (LR) or without AR (LNR) and from healthy volunteers without rhinitis (HNR). A total of 5980 DEGs were detected in the LR vs. HNR group, 4888 DEGs in the LR vs. LNR group, and 4233 DEGs in the LNR vs. HNR group (**Supplementary Figure S4**). AM-associated genes were set by combining the DEGs from the LR vs. LNR group and LR vs. HNR group and then removing the DEGs from the LNR vs. HNR group using a Venn diagram (**Figure 4A**). A total of 1932 AM-associated genes were obtained. KEGG analysis was employed to explore the potential biological function of these genes. As shown in **Figure 4B**, cytokine-cytokine receptor interaction was identified as the most significant pathway.

As shown in **Figure 4C**, 53 genes were enriched in cytokine-cytokine receptor interactions. The expression levels of those genes were further detected in the microarray of AM. As shown in **Figure 4D**, the expression levels of CCL1, CCL20, CCL22, EDAR, IL1B, IL34, and IL4R in the AM group were significantly different from those in the HS group. Among them, the mRNA and protein levels of IL4R and IL17A were significantly increased in the skin of the LR group compared to those of the HNR and LNR groups (**Figures 4E, F**). In addition, the ELISA results found that serum IL17A and IL4R were upregulated in the LR group compared to the HNR and LNR groups (**Figure 4G**). Subsequently, correlation analysis revealed that the mRNA expression levels of IL17A and IL4R in LR lesions were significantly and positively related to TNSS, and the IL17A expression level showed a closer and more significant association with TNSS than the IL4R expression level did (**Figure 4H**).

### Decreased Expression of Genes Involved in Gland Secretion Contributes to Atopic March

**Figure 5:**
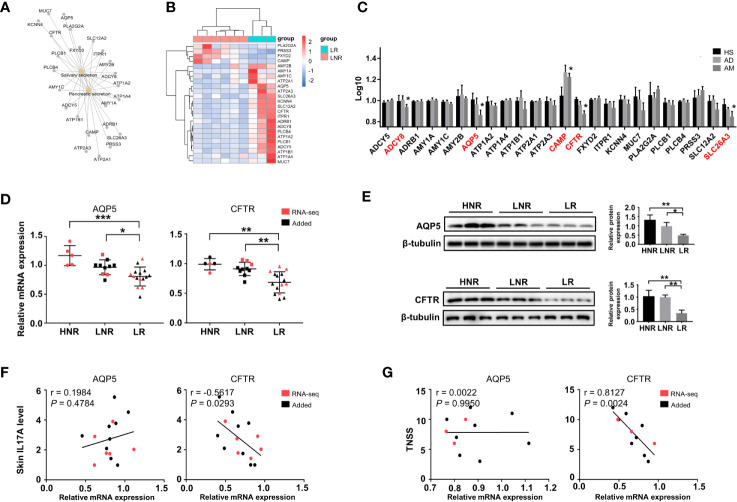
Altered expression of genes involved in gland secretion identification in lesions of AD patients with atopic march. **(A)** The interaction network of salivary and pancreatic secretion and the DEGs involved. **(B)** Sample cluster analysis based on the expression pattern of the genes enriched in salivary and pancreatic secretion [LNR group (n = 3), LR group (n = 6)]. **(C)** The expression level of DEGs enriched in gland secretion in the microarray of AM [HS group (n = 3), AD group (n = 4), AM group (n = 3]. **(D)** The mRNA expression levels of AQP5 and CFTR in the skin [HNR group (n = 5), LNR group (n = 11), LR group (n = 15)]. **(E)** The protein expression levels and quantitative analysis of AQP5 and CFTR in the skin [HNR group (n = 3), LNR group (n = 3), LR group (n = 3)]. **(F)** The correlation of AQP5 and CFTR with skin IL17A levels (n = 15). **(G)** The correlation of AQP5 and CFTR with TNSS (n = 11). *P < 0.05, **P < 0.01, ***P < 0.001 according to one-way ANOVA followed by Tukey’s or Dunnett’s multiple comparisons.

Interestingly, significant changes in salivary secretion and pancreatic secretion were also identified by KEGG analysis (**Figure 4B**), in which a total of 24 genes were involved. These genes were also considered AM-associated genes (**Figure 5A**). Interestingly, we supposed that the expression pattern of those genes could distinguish LR from LNR (**Figure 5B**) via cluster analysis. Among the 24 genes, five genes displayed remarkably aberrant expression in the AM group compared to the HS group (**Figure 5C**).

The qRT-PCR and Western blotting results confirmed that AQP5 and CFTR were significantly downregulated in the LR group compared to those in the HNR and LNR groups (**Figures 5D, E**). Correlation analysis illustrated that the CFTR mRNA expression level was significantly and negatively associated with skin IL17A level and TNSS, while no remarkable relationship was found between AQP5 and skin IL17A level and TNSS (**Figures 5F, G**).

## Discussion

AD affects up to 20% of children and 2-3% of adults worldwide, in which the Chinese AD patient population may be the largest ethnic AD population (∼9% in adults) (14, 15). The heterogeneity of Chinese AD patients remains poorly understood. Compared with previous studies on Chinese patients (6, 16), our study conducted more precise stratifications based on phenotypes and showed marked Th17 activation in EAD rather than IAD. Moreover, the expression of *IL17A*, *IL4R*, *AQP5*, and *CFTR* was associated with AM in the Chinese Han population by functional analyses of the transcriptome and further experiments.

It is well established that AD is characterized by Th2 bias, and elevated Th17 level distinguish Asian AD patients from European AD patients (1, 6). However, increased IL17 signaling pathway also was found in European IAD patients (17). Our results further demonstrated that the enhanced IL17 signaling pathway was significant in Chinese EAD patients rather than in IAD patients, indicating that not all Chinese AD patient had high IL17 level. Furthermore, correlation analysis revealed that IL17A was positively associated with IgE level and AD severity in EAD. IgE is produced by B cells in the Th2-biased cytokine microenvironment and represents a good cumulative readout of systemic Th2 bias (18). However, recent studies reported that B cells could also express IL17RA, and IL17 plays an important role in Th2 differentiation and IgE response (19). In line with these notions, IL17A might function as an enhancer of Th2 cytokine and serum IgE production in Chinese EAD patients (20).

Besides, we analyzed the genes related to the AM in the Chinese Han population. The results showed that patients with AM exhibited higher IL17A and IL4R levels both in the skin and serum with a significantly positive correlation with AR severity (TNSS), indicating that the Th2/Th17 axis might contribute to AM. Interestingly, *IL17A* showed a more significant association with TNSS than *IL4R* in Han Chinese patients and has been considered a risk allele gene related to AM by GWAS (21). A recent study revealed that IL17A could induce nasal fibroblasts to produce thymic stromal lymphopoietin (TSLP), which promotes Th2 inflammation in AR (22). In addition, IL17A could regulate DC migration to the peribronchial lymph nodes and allergen presentation in experimental allergic asthma (23). These findings implied a close relationship between IL17A and AR in the Th2-biased cytokine microenvironment. Given that Asian patients show higher IL17 than other races, whether IL17A displays a closer correlation with AR than IL4R in other races needs further investigation.

In addition to abnormal cytokine-cytokine receptor interactions, our results revealed a remarkable transcriptomic alteration in gland secretion in lesional skin from AD patients with AR compared to those without AR. Indeed, dysfunction of the sweat gland was reported in some AD patients (24). This leads the skin with impaired barrier function to be more sensitive to antigens, and sweat containing inflammatory cytokines could initiate inflammation at points of allergen entry (24). In addition, *AQP5* and *CFTR* were identified as AM-associated genes in our study. AQP5 is an essential factor that can modulate the fluid content of the upper airway and nasopharyngeal secretions(Skowron-zwarg et al., 2007). In the skin, AQP5 is mainly located in the luminal and basolateral surfaces of eccrine secretory cells in sweat glands (25). Although it was significantly decreased in the lesions of AD patients with AR, its expression level was barely related to serum IL17A or rhinitis severity. However, the downregulated CFTR in the lesions of AD patients with AR was negatively correlated with serum IL17A and TNSS. As an apical membrane anion channel, CFTR regulates fluid homeostasis in both the airway and sweat glands (26). It was reported that chemokines induced by IL17 were enhanced in the absence of CFTR activity in human airway epithelial cells (27). Thus, we speculated that a low level of CFTR in the lesion might amplify the IL17A-induced response contributing to AM.

Recently, it has been proposed that AD and psoriasis could be considered diseases occurring across a spectrum, with certain subtypes having overlapping characteristics (28). Anti-IL17 treatment seems to be promising for atopic patients (1, 29). However, some psoriasis patients present eczematous reactions with atopic dermatitis-like features after receiving IL17 antibody treatment (30). This implied that inhibition of Th17 alone might tilt the balance toward the Th2 axis. Therefore, although our study highlighted the role of IL17A in EAD and AM, anti-Th17 therapy for AD should be made cautiously.

There were some limitations in our study. The sample size was small and obtained from only two regions of China. Given that the source of human samples is influenced by many factors, such as region, environment, climate, diet, and allergen, a larger-scale study with an extended sample size needs to be conducted to represent the average characteristics of AD among the Chinese Han population. In addition, the sample from other races needs to be studied to describe national heterogeneity.

In conclusion, our research characterized the contribution of IL17A in EAD and AM in the Chinese Han population. Furthermore, abnormal expression of genes involved in gland secretion in lesions might contribute to the development of AM. This finding partially explained the phenotypic differences of AD subtypes and provided potential references for phenotype-specific therapies.

## Data Availability Statement

The data presented in the study are deposited in the NCBI Bioproject repository, accession number Bioproject ID: PRJNA691738.

## Ethics Statement

The studies involving human participants were reviewed and approved by ethics committee of Guangzhou Institute of Dermatology (201803). Written informed consent to participate in this study was provided by the participants’ legal guardian/next of kin.

## Author Contributions

YL, ZW, and XZ: study design. XT and YL: data collection. BL, LC, YX, JW, and JL: analysis of data. YY and LS: interpretation of data. ZW, BL, and JZ: drafting the article. All authors contributed to the article and approved the submitted version.

## Funding

This work was supported by the Health Science and Technology Project of Guangzhou (20191A011070), the Science and Technology Plan Project of Guangzhou (201904010082), the Guangdong science and technology research fund project (B2020068), and the National Natural Science Foundation of China (grant nos. 81773310).

## Conflict of Interest

The authors declare that the research was conducted in the absence of any commercial or financial relationships that could be construed as a potential conflict of interest.
