# Supplementary material for: RNA-Seq Identifies Marked Th17 Cell Activation and Altered CFTR Expression in Different Atopic Dermatitis Subtypes in Chinese Han Populations
Source: Front Immunol. 2021 Apr 1;12:628512. doi: 10.3389/fimmu.2021.628512 (PMC8047326; doi:10.3389/fimmu.2021.628512)
Supplement: Supplementary file 1 [file DataSheet_1.docx]

**Supplementary Table S1. Details of patients enrolled in the study.**

|  | **HS** | **IAD** | **EAD** |
| --- | --- | --- | --- |
| Total (n) | 8 | 10 | 18 |
| Male | 4 | 5 | 10 |
| Female | 4 | 5 | 8 |
| Region (Guangzhou/Shanghai) | 4/4 | 5/5 | 10/8 |
| Age (mean ± SD) | 21.00 ± 5.23 | 20.17 ± 4.41 | 20.30 ± 6.57 |
| With rhinitis | 3 | 3 | 8 |
| SCORAD | / | 42.33 ± 13.00 | 39.40 ± 15.17 |

**Supplementary Table S2. Details of patients enrolled in RNA-seq.**

| **ID** | **Gender** | **Age** | **Region** | **SCORAD score** | **Rhinitis** | **Type** |
| --- | --- | --- | --- | --- | --- | --- |
| P1 | Female | 25 | Guangzhou | 56 | with | Intrinsic AD |
| P2 | Female | 23 | Guangzhou | 57.9 | without | Extrinsic AD |
| P3 | Male | 22 | Guangzhou | 39 | with | Extrinsic AD |
| P4 | Male | 18 | Guangzhou | 46 | with | Extrinsic AD |
| P5 | Male | 29 | Shanghai | 42 | without | Intrinsic AD |
| P6 | Male | 17 | Guangzhou | 51 | without | Extrinsic AD |
| P7 | Male | 20 | Shanghai | 35 | with | Extrinsic AD |
| P8 | Female | 9 | Shanghai | 58 | with | Extrinsic AD |
| P9 | Male | 22 | Shanghai | 46 | with | Extrinsic AD |
| P10 | Male | 20 | Shanghai | 35 | unknown | Extrinsic AD |
| H1 | Female | 28 | Guangzhou | / | with | Health |
| H2 | Female | 24 | Guangzhou | / | with | Health |
| H3 | Male | 23 | Guangzhou | / | without | Health |
| H4 | Female | 21 | Shanghai | / | without | Health |
| H5 | Male | 10 | Shanghai | / | with | Health |

**Supplementary Table S3. Primer sequences used for RT-PCR.**

| **Primer name** | **Sequence (5'-3')** |
| --- | --- |
| PPARG FW | ACCAAAGTGCAATCAAAGTGGA |
| PPARG RV | ATGAGGGAGTTGGAAGGCTCT |
| IL17A FW | AGATTACTACAACCGATCCACCT |
| IL17A RV | GGGGACAGAGTTCATGTGGTA |
| IL2 FW | TCCTGTCTTGCATTGCACTAAG |
| IL2 RV | CATCCTGGTGAGTTTGGGATTC |
| CXCL10 FW | GTGGCATTCAAGGAGTACCTC |
| CXCL10 RV | TGATGGCCTTCGATTCTGGATT |
| IL13 FW | CCTCATGGCGCTTTTGTTGAC |
| IL13 RV | TCTGGTTCTGGGTGATGTTGA |
| IL22 FW | GCTTGACAAGTCCAACTTCCA |
| IL22 RV | GCTCACTCATACTGACTCCGT |
| IL9 FW | CTCTGTTTGGGCATTCCCTCT |
| IL9 RV | GGGTATCTTGTTTGCATGGTGG |
| IL4R FW | CGTGGTCAGTGCGGATAACTA |
| IL4R RV | TGGTGTGAACTGTCAGGTTTC |
| AQP5 FW | GCCACCTTGTCGGAATCTACT |
| AQP5 RV | GGCTCATACGTGCCTTTGATG |
| CFTR FW | AAAAGGCCAGCGTTGTCTCC |
| CFTR RV | AAACATCGCCGAAGGGCATTA |
| GAPDH FW | CATGTACGTTGCTATCCAGGC |
| GAPDH RV | CTCCTTAATGTCACGCACGAT |

**Supplementary Table S4. Antibodies used for western blot analysis.**

| **Antibodies** | **Product code** |
| --- | --- |
| Anti-NLRP3 | ab260017 |
| Anti-ICAM1 | ab53013 |
| Anti-AQP5 | ab92320 |
| Anti-CFTR | ab181782 |
| Anti-PPAR-delta | ab178866 |
| Anti-β-tubulin | sc-166729 |
| Anti-GAPDH | sc-47724 |

**Supplementary Figures and Figure Legends**

**Supplementary Figure S1**


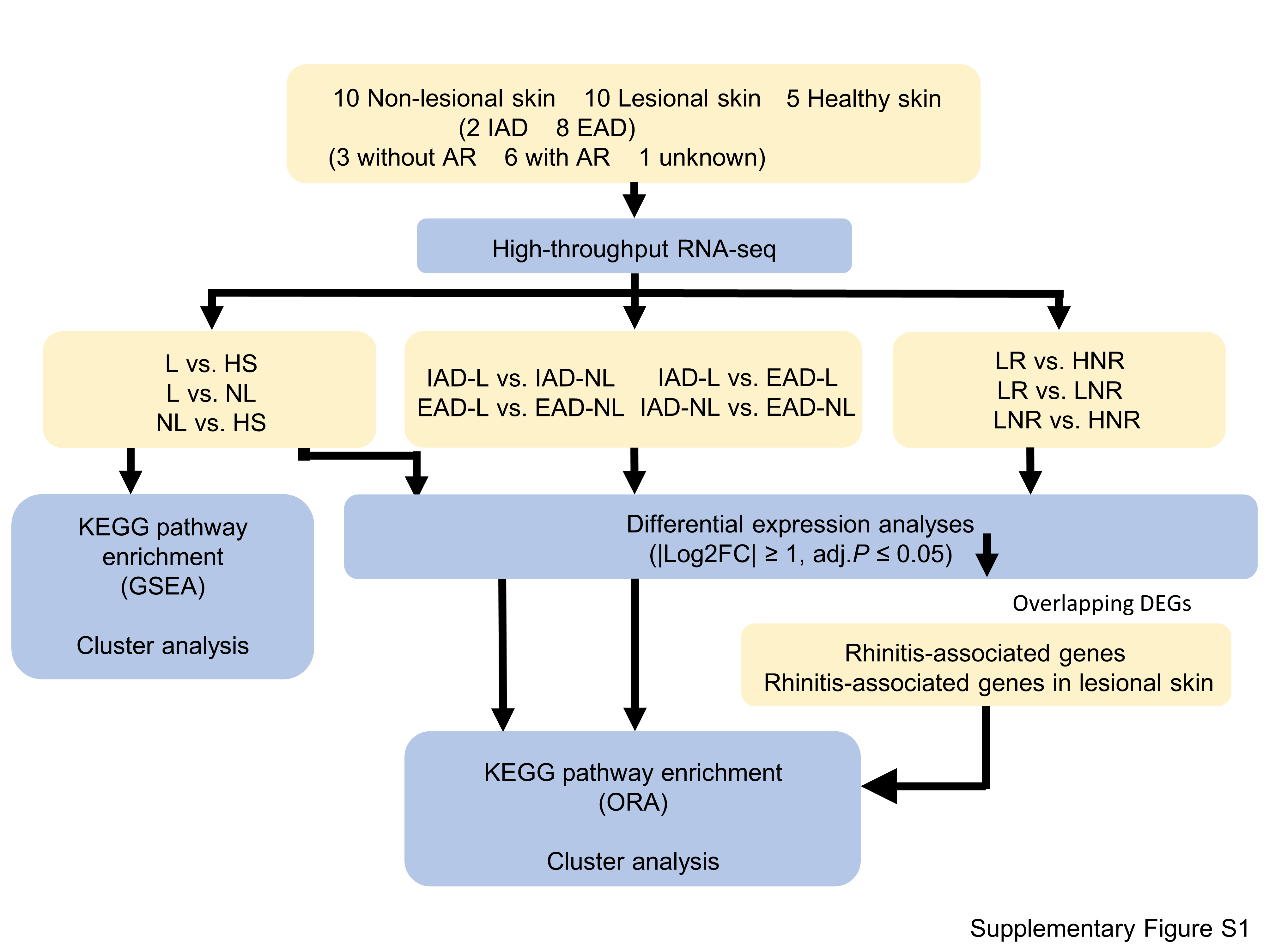


**Supplementary Figure S1. Work Flowchart of RNA-seq analysis.**

IAD: intrinsic AD, EAD: extrinsic AD, AR: allergic rhinitis, L: lesional skin, NL:non-lesional skin, H:normal skin from healthy volunteers, LR: lesional skin samples from AD patients with rhinitis, LNR: lesional skin samples from AD patients without rhinitis, HNR: healthy volunteer without rhinitis, GSEA: gene set enrichment analysis, DEGs: differentially expressed genes.

**Supplementary Figure S2**


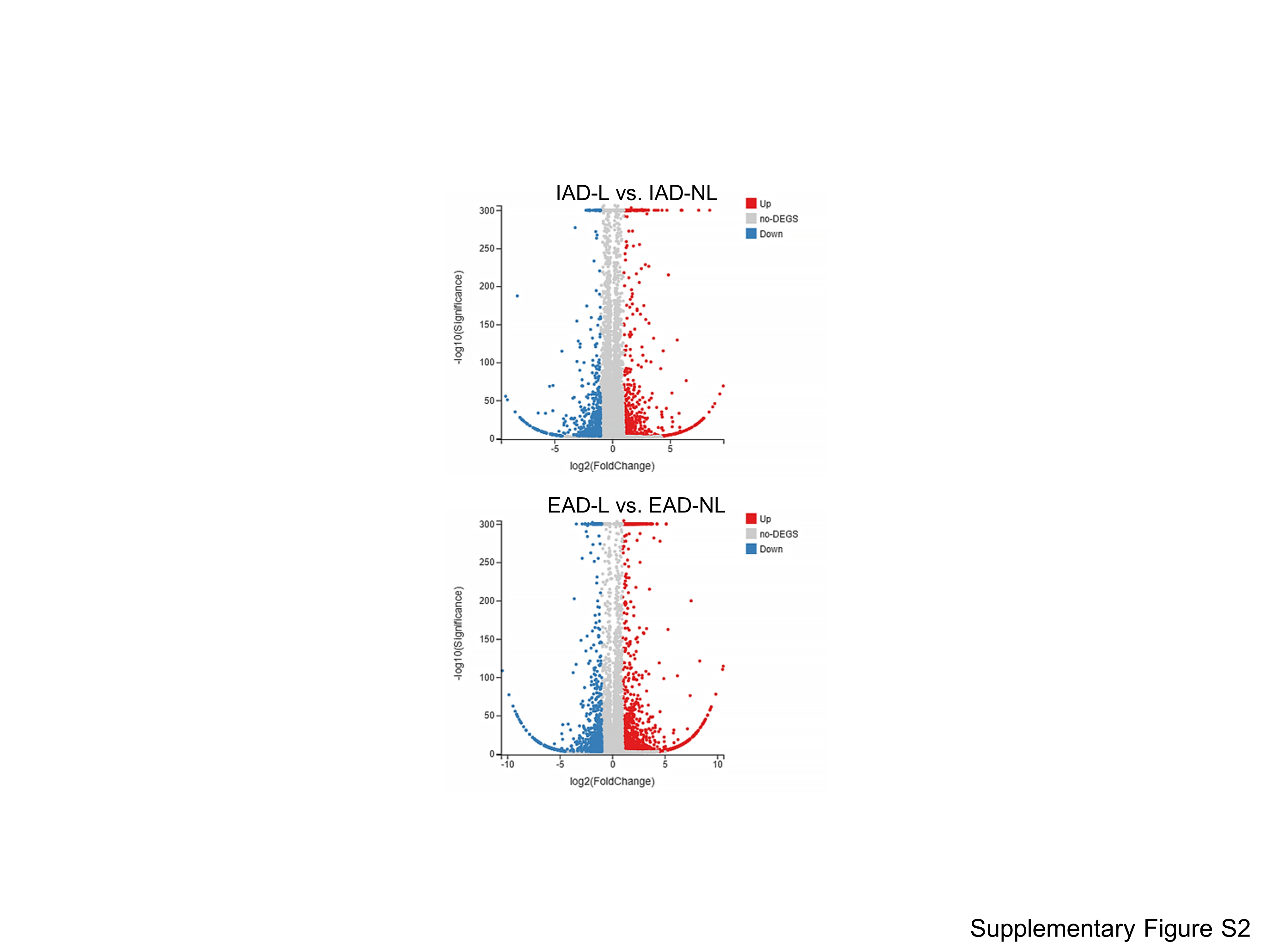


**Supplementary Figure S2. DEGs identification in Chinese intrinsic AD and extrinsic AD.**

Volcano plot of DEGs between IAD-L (n = 2) and IAD-NL group (n = 2) and between EAD-L (n = 8) and EAD-NL group (n = 8).

**Supplementary Figure S3**


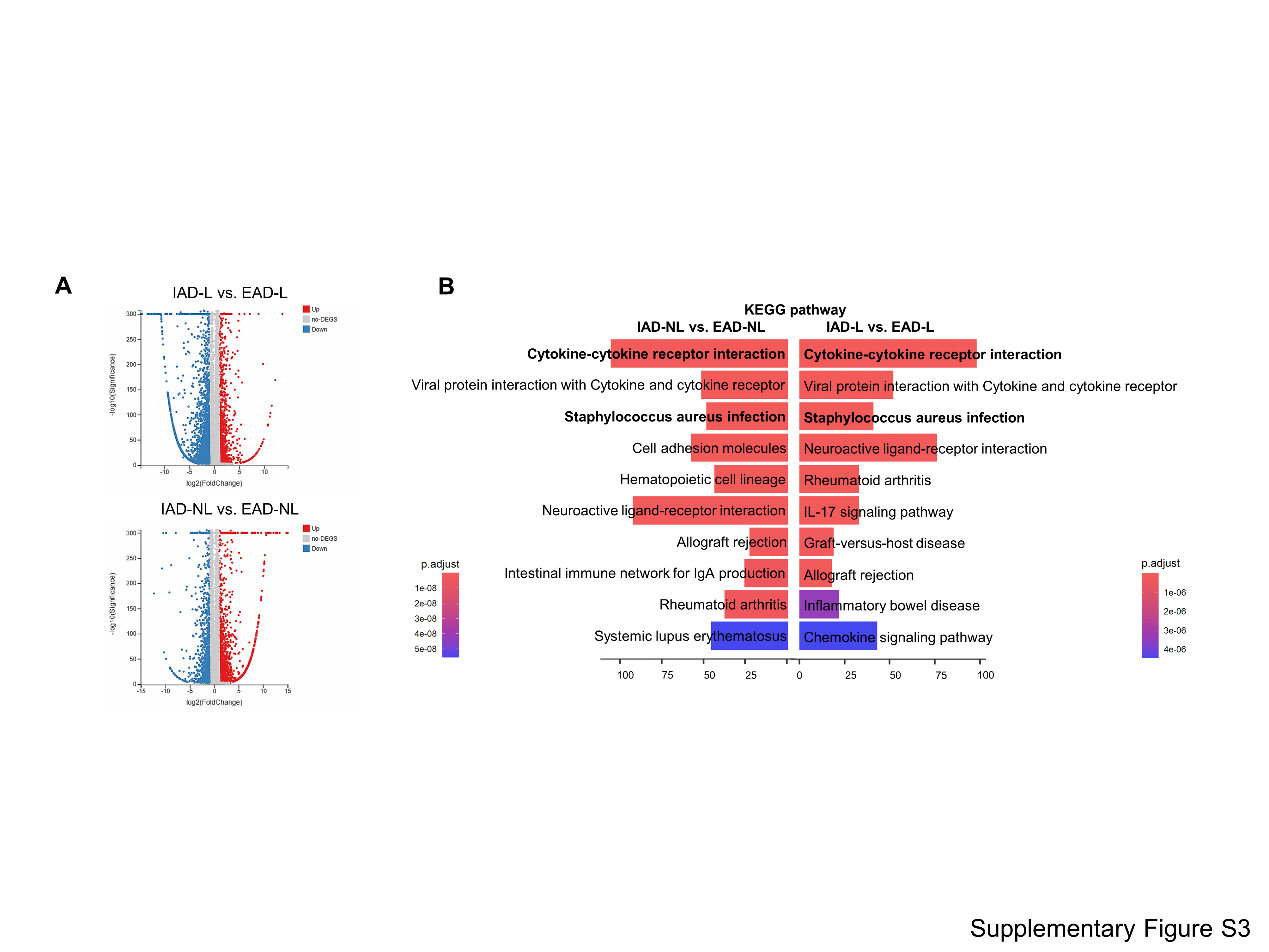


**Supplementary Figure S3. DEGs identification and function enrichment analyses of IAD-L vs. EAD-L and IAD-NL vs. EAD-NL.**

**(A)** Volcano plot of DEGs between IAD-L (n = 2) and EAD-L (n = 8), and between IAD-NL (n = 2) and EAD-NL (n = 8). **(B)** Top 10 enriched KEGG pathways. (*P* <0.05)

**Supplementary Figure S4**


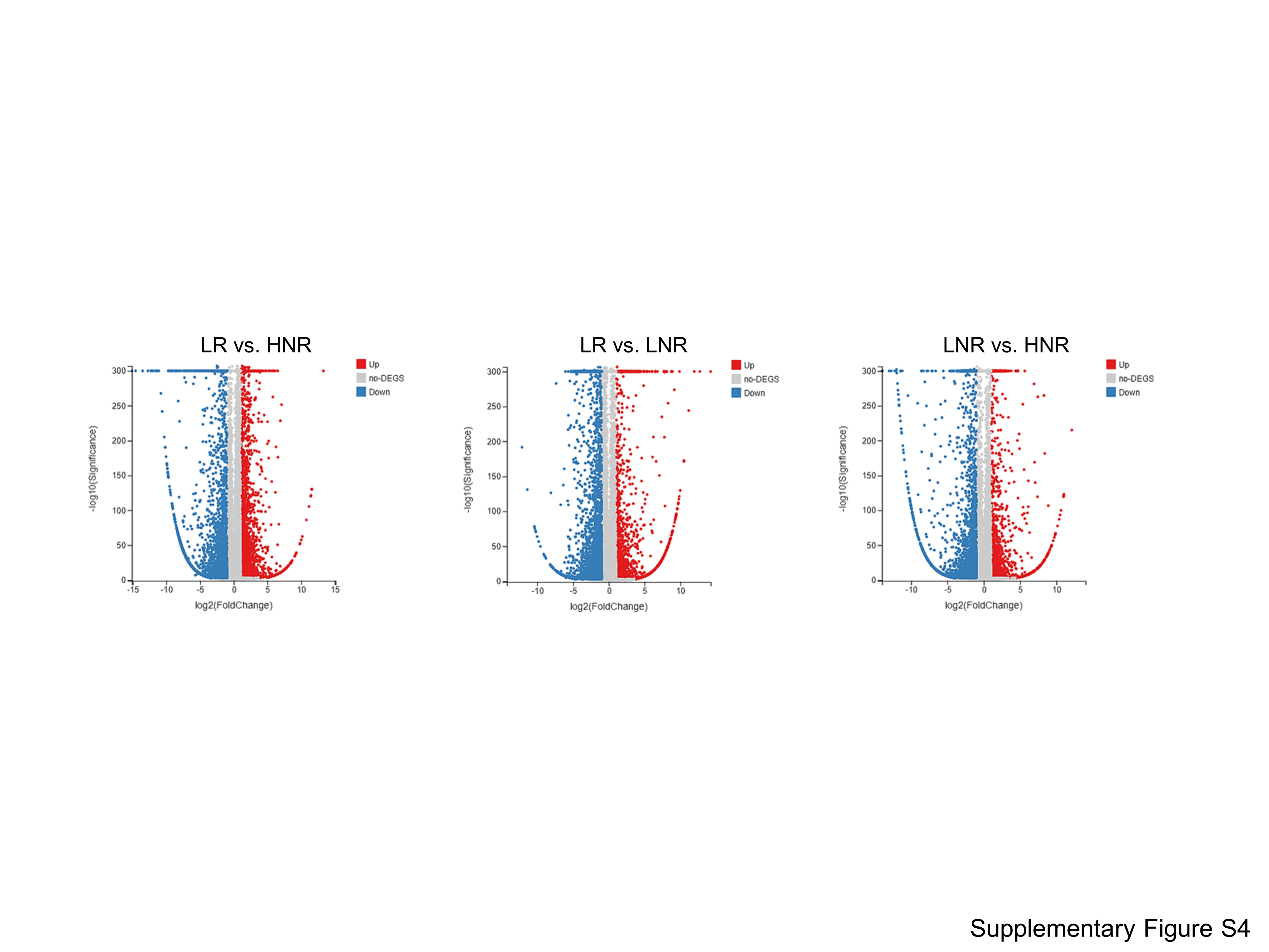


**Supplementary Figure S4. DEGs identification among sample subgroups related to rhinitis.**

Volcano plot of DEGs between LR (n = 6) and HNR (n = 2), between LR (n = 6) and LNR (n = 3), and between LNR (n = 3) and HNR (n = 2).
